# Accuracy of a markerless motion capture system in estimating upper extremity kinematics during boxing

**DOI:** 10.3389/fspor.2022.939980

**Published:** 2022-07-25

**Authors:** Bhrigu K. Lahkar, Antoine Muller, Raphaël Dumas, Lionel Reveret, Thomas Robert

**Affiliations:** ^1^Univ Lyon, Univ Gustave Eiffel, Univ Claude Bernard Lyon 1, LBMC UMR_T9406, Lyon, France; ^2^INRIA Grenoble Rhone-Alpes, LJK, UMR 5224, Grenoble, France

**Keywords:** markerless vs. marker-based, kinematic analysis, evaluation, elite sport, upper-limb, sports-performance

## Abstract

Kinematic analysis of the upper extremity can be useful to assess the performance and skill levels of athletes during combat sports such as boxing. Although marker-based approach is widely used to obtain kinematic data, it is not suitable for “in the field” activities, i.e., when performed outside the laboratory environment. Markerless video-based systems along with deep learning-based pose estimation algorithms show great potential for estimating skeletal kinematics. However, applicability of these systems in assessing upper-limb kinematics remains unexplored in highly dynamic activities. This study aimed to assess kinematics of the upper limb estimated with a markerless motion capture system (2D video cameras along with commercially available pose estimation software Theia3D) compared to those measured with marker-based system during “in the field” boxing. A total of three elite boxers equipped with retroreflective markers were instructed to perform specific sequences of shadow boxing trials. Their movements were simultaneously recorded with 12 optoelectronic and 10 video cameras, providing synchronized data to be processed further for comparison. Comparative assessment showed higher differences in 3D joint center positions at the elbow (more than 3 cm) compared to the shoulder and wrist (<2.5 cm). In the case of joint angles, relatively weaker agreement was observed along internal/external rotation. The shoulder joint revealed better performance across all the joints. Segment velocities displayed good-to-excellent agreement across all the segments. Overall, segment velocities exhibited better performance compared to joint angles. The findings indicate that, given the practicality of markerless motion capture system, it can be a promising alternative to analyze sports-performance.

## Introduction

Boxing is an intensive combat sport, involving highly dynamic and non-symmetrical movements of the front and rear arms with the role of attack or defense as situation demands. In such sports, high-performance athletes are often characterized by their agility, i.e., the ability to punch or evade swiftly by maintaining fluidity of motion. To achieve a powerful punch during offensive action and quick retraction during defense, coordination of the body segments plays a vital role (Dinu and Louis, 2020). As body segments' coordination is often a consequence of how the adjacent segments are oriented to each other (Zajac and Winters, 1990; Putnam, 1993), estimating segment pose (positions and orientations) during boxing may be helpful to analyze the performance athletes. Furthermore, the velocities at which body segments move and coordinate with each other have been reported to vary across athletes based on their skills (Putnam, 1993). Therefore, estimating velocities of the body segments seems essential to analyze sports-performance during boxing.

To quantify body segment kinematic variables, marker-based motion capture has been most widely used. In such systems, skin markers are placed on the specific anatomical landmarks, based on which body segment coordinate systems are defined to estimate 3D pose of the segments. While marker-based methods are traditionally referred to as standard, they are commonly performed in a laboratory environment and require adequate skills in physical palpation of landmarks. Even with necessary skills, such palpation is examiner-dependent and at times tends to produce systemic bias for an examiner (Johnson et al., 2018). Furthermore, joint kinematics are also largely affected by soft tissue artifact (Camomilla et al., 2017; Lahkar et al., 2021). Alternatively, measurements based on wearable sensors such as inertial measurement units have been recently shown effective in natural environment in estimating joint angles of the lower extremity with moderate to strong accuracy (Al Borno et al., 2022). Studies also presented the use of inertial measurement units in estimating hand velocity (Kimm and Thiel, 2015; Punchihewa et al., 2020) and other body segments (Dinu and Louis, 2020) during a sport activity. While such studies are useful for understanding differences in skills between athlete groups, placing sensors or markers on the body surface may be inconvenient and potentially distracting to an athlete and practically impossible during a live combat.

With the rapid advancement of computer vision research, human movement study has received a significant stride allowing unobtrusive capture of data using video-based markerless motion capture (Colyer et al., 2018; Armitano-Lago et al., 2022). These methods rely on 2D video data combining with generative or discriminative algorithms to estimate human pose in 3D (Colyer et al., 2018). Generative approach often involves fitting a predefined model of the subject to 2D visual cues such as image features from detectors or to 3D cues such as a visual hull reconstruction with the help of silhouette matching algorithms (Corazza et al., 2006, 2007; El-Sallam et al., 2013). On the other hand, learning-based discriminative algorithms, particularly deep neural network, involves detecting sparse set of learned features such as joint key points describing a subject's pose in 2D. In this family, openly accessible pose estimator like OpenPose (Cao et al., 2021) has received significant attention in human movement analysis and similarly DeepLabCut (Mathis et al., 2018) for both human and non-human activities. As these tools are primarily intended for 2D pose estimation, some studies leveraged its potential in estimating 2D kinematics of the lower limb (hip, knee, and ankle joint) during gait (Stenum et al., 2021), vertical jump (Drazan et al., 2021), and under water running (Cronin et al., 2019). Progressing further, others focused on estimating 3D poses from 2D images of multiple calibrated cameras using triangulation during walking (Nakano et al., 2020; Needham et al., 2021; Pagnon et al., 2022), jumping (Nakano et al., 2020; Needham et al., 2021), running (Needham et al., 2021; Pagnon et al., 2022), cycling (Pagnon et al., 2022), and throwing (Nakano et al., 2020). While these studies demonstrated the potential of openly accessible pose estimation tools in estimating 3D joint kinematics, most of them primarily evaluated the lower extremity. As far as we are aware of Nakano et al. (2020) estimated 3D joint positions of the shoulder, elbow, and wrist and evaluated against traditional marker-based approach during walking, jumping, and ball throwing activity. A mean absolute error up to 4 cm was observed at the wrist, 4.7 cm at the elbow, and 2.2 cm at the shoulder during throwing activity.

In a recent development in markerless video-based systems, Theia3D (Theia Markerless, Inc., Kingston, Ontario) has emerged as a rapidly evolving commercial pose estimation software. The software implements deep convolutional neural network combining with standard biomechanical pose estimation approaches (inverse kinematics) to estimate 3D pose of human body segments. Using this tool, studies showed decent kinematic accuracies compared to marker-based method while maintaining good repeatability both in laboratory environment (Kanko et al., 2021a,b) and in community settings (Mcguirk et al., 2022; Riazati et al., 2022). These studies, however, mainly provide the assessment of the lower extremity kinematics during either treadmill or over ground walking activity.

While it is relevant to evaluate the usability of markerless systems in a highly dynamic and non-symmetrical sport such as boxing, it still remains unknown how accurate these systems are in estimating upper-limb kinematics as compared to marker-based approach. This study aimed to assess whether a markerless approach (use of video cameras + commercial pose estimation software Theia3D) can be used to estimate upper-limb kinematics as an alternative to the state-of-the-art marker-based approach for sports-performance analysis during “in the field” boxing.

## Materials and methods

### Participants

A total of three elite boxers volunteered in the study at the boxing arena of National Institute of Sport, Expertise, and Performance (INSEP, Paris, France). Out of the three boxers, one is competing at the national level and two others at the international level. All of them are undergoing regular training at INSEP for Paris Olympics, 2024. Demographic details of the athletes are presented in Table 1. The athletes, after being fully informed about the objectives and protocol of the study, signed an informed consent form. The study and the procedures were approved by an institutional review board.

**Table 1:** Demographic details of the athletes.

**Athlete**	**Gender**	**Age (years)**	**Height (m)**	**Body mass (kg)**
1	Male	20	1.72	54
2	Male	18	1.90	78
3	Female	19	1.63	59

### Data acquisition setup and protocol

**Figure 1:**
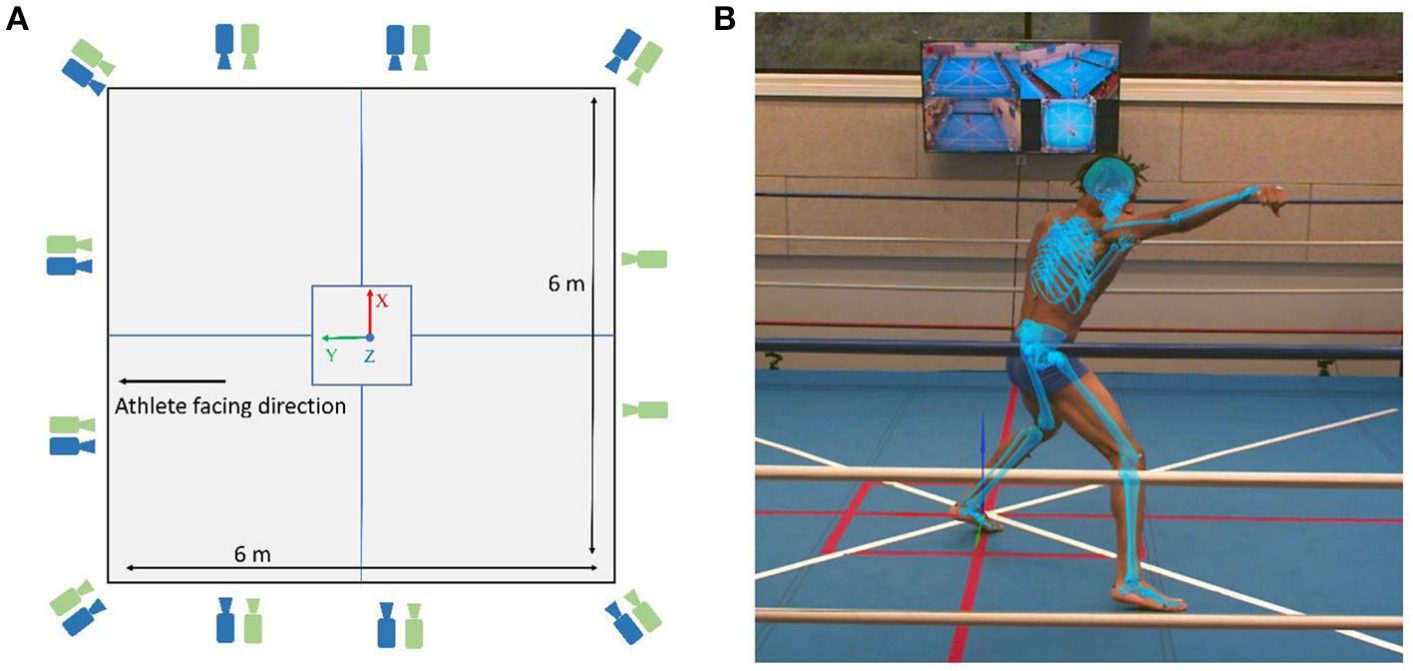
**(A)** Layout of the boxing ring with green and blue cameras depicting optoelectronic and video cameras, respectively. **(B)** An example of the boxing punch with Theia3D multibody model overlaid on the 2D video image.

Boxing data were collected synchronously using an optoelectronic marker-based system (12 Qualisys Miqus and Arqus cameras; 2–5 megapixel) at 300 Hz, and using a markerless 2D video-based system (10 Qualisys Miqus video cameras; 2 megapixel) at 60 Hz. Both the types of cameras, optoelectronic and video, were placed next to each other as a pair around the boxing ring, except two optoelectronic cameras placed separately to the posterior aspect of the athlete (Figure 1A). All the cameras were connected to Qualisys Track Manager for allowing them to be synchronized and calibrated in space and time, giving a single global reference frame nearly at the center of the boxing platform. Camera setup and placement was performed by the team members with expertise in both optoelectronic and video-based motion analysis. Specific attention was provided to the 2D video-based cameras to comply with recommended specifications for resolution, focus, and exposure time.

Prior to the sessions, the boxers were outfitted with 44 retro-reflective skin markers placed by a single operator with adequate palpation skills on the relevant landmarks of the whole body (Wu et al., 2002, 2005). The details of the marker-set and their anatomical locations are provided in the Supplementary Material. A professional coach instructed each boxer to perform specific five shadow boxing trials of different characteristics, with 4–5 repetitions in each trial (Table 2). In between repetitions within a trial, boxers were instructed to perform footwork and remain in defensive pose with elbow flexed guarding their body and face, as classically performed during a contest.

**Table 2:** Boxing trials and their specific characteristics.

**Trial**	**Characteristics**
1	Direct from the front arm to the face
2	Direct from the front arm to the body
3	Double of the front arm to the face
4	Rear arm jab + front arm hook
5	Front arm (uppercut to the body + hook to the body + hook to the face)

### Data processing and analysis

#### Multibody models

Theia3D embedded multibody kinematic model consists of two separate kinematic chains: one for the lower extremity and one for the upper extremity, and a separate head segment with six degrees of freedom (DoFs) (https://www.theiamarkerless.ca/docs/model.html). In this study, we will only adhere to the upper extremity model in the following descriptions. The upper extremity chain comprises the thorax as root segment with six DoFs with respect to the ground, followed by the clavicle, upper arm, forearm, and hand segments bilaterally. The clavicle, at its proximal end, is connected to the thorax with a two rotational DoFs constraint, while distally connected to the upper arm with a three rotational DoFs at the shoulder joint. The elbow and the wrist joints are constrained to have two DoFs, restricting abduction/adduction (Abd/Add) at the elbow and internal/external (Int/Ext) rotation at the wrist.

For the marker-based multibody model, a gender-specific generic template was created in Visual3D (C-motion, Germantown, USA, v2021.11.3) to have identical body segments and joint constraints as that of the Theia3D model. The shoulder, cervical, lumbar, and thoracic joint centers were defined based on the regression equations adopted from the study of Dumas and Wojtusch (2018). The midpoint between the medial and lateral humeral epicondyles was defined as the elbow joint center and the midpoint between the ulnar and radius styloid processes as the wrist joint center. Segment reference frames were defined following the methodology reported in the study of Dumas and Wojtusch (2018).

For both the models, the center of mass position for each segment was defined according to the study of Dumas and Wojtusch (2018).

#### Kinematic estimation

Markerless motion capture data were processed with Theia3D (v2021.2), a deep learning-based software. The underlying principle of the software is detailed elsewhere in the study of Kanko et al. (2021a) and briefly delineated hereafter. The software relies on synchronized and calibrated videos as input that uses pre-trained deep convolutional neural networks to estimate 2D positions of learned key features (e.g., joint locations and surface landmarks) within the frames of video data, thus enabling to obtain the features in 3D space. The embedded multibody kinematic model is adapted to fit 3D subject-specific features, and a multibody kinematic optimization scheme (Begon et al., 2018) allows to perform 3D pose estimation during an activity. In this study, estimated 3D poses (4-by-4 matrices) of the body segments were exported to Visual3D to compute joint kinematics and segment velocities. Figure 1B illustrates an example of the Theia3D model obtained with multibody kinematic optimization during boxing.

Regarding the marker-based data, the generic multibody template was adapted to obtain subject-specific scaled models, and segment's pose estimation throughout all motion frames was obtained using multibody kinematic optimization (Begon et al., 2018) within Visual3D. Proper segment-specific marker weights were implemented and tuned based on residual analysis, with highest weight at the thorax, followed by the upper arm, forearm, and hand.

The markerless vs. marker-based method was assessed by the following kinematic variables: joint center positions, joint angles, and linear segment velocities. The joint center positions at the shoulder, elbow, and wrist were retrieved from the pose matrices resulting from multibody kinematic optimization. Then, 3D Euclidean distances between corresponding joint centers across all the trials and subjects were computed. The joint angles at the shoulder (between thorax and upper arm), elbow (between upper arm and forearm), and wrist (between forearm and hand) were computed with cardan sequences of rotation adopted from the study of Wu et al. (2002). Linear segment velocity magnitudes were derived from the center of mass positions of each segment in the global reference frame. The kinematic variables were exported to MATLAB (MathWorks, USA), and a 4th-order low-pass Butterworth filter was implemented to filter both the joint angles and segment velocities with cutoff frequency of 8 Hz.

#### Statistical analysis

The deviation between corresponding joint centers estimated with marker-based and markerless system was assessed as mean (standard deviation) or median (interquartile range) based on normality outcomes across all the trials and subjects. The degree of agreement between joint angles resulting from both the methods was assessed using Bland–Altman analysis (Bland and Altman, 1986). Bias (*b*), confidence interval (*CI*; 1.96 times standard deviation or 1.45 times interquartile range for non-normal distributions), coefficient of determination (*R*^2^), and root mean square difference (*RMSD*) were calculated for comparison. The same statistical parameters were used for comparing segment velocity magnitudes. All the analyses were performed for the front and rear limbs separately using customized MATLAB routines.

## Results

### Joint center positions

**Figure 2:**
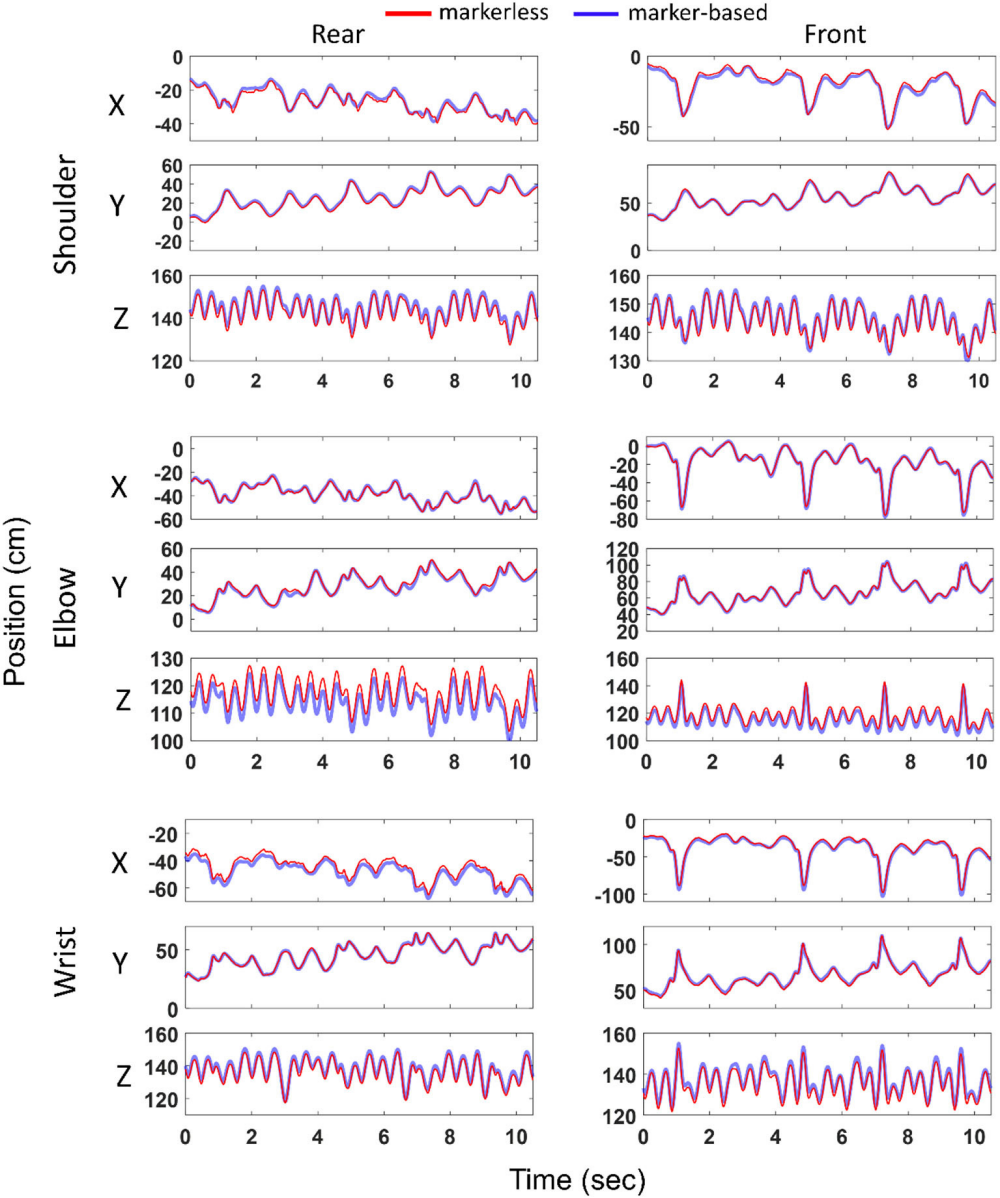
Joint center positions (ordinate) at the shoulder, elbow and wrist in the global reference frame (*X, Y, Z*) computed with marker-based and markerless methods and represented over time (abscissa). Left and right columns represent joints of the rear and front limbs, respectively. Example shown for the second athlete and first boxing trial. Blue and red colors represent marker-based and markerless joint center positions, respectively.

An example (second athlete and first trial) of the joint center positions in the global reference frame estimated with markerless vis-à-vis marker-based approach is presented in Figure 2. Overall across all the subjects and trials, the joints of the front and rear limbs followed similar trajectories measured with both the systems. Differences [median (interquartile range)] between markerless and marker-based joint centers for the front shoulder, elbow, and wrist were found as 2.3 (0.8), 3.1 (0.8), and 1.8 (1.2) cm, respectively. These values for its rear counterparts were 2.3 (1.3), 3.1 (0.9), and 2.2 (2.5) cm, respectively.

### Joint angles

**Figure 3:**
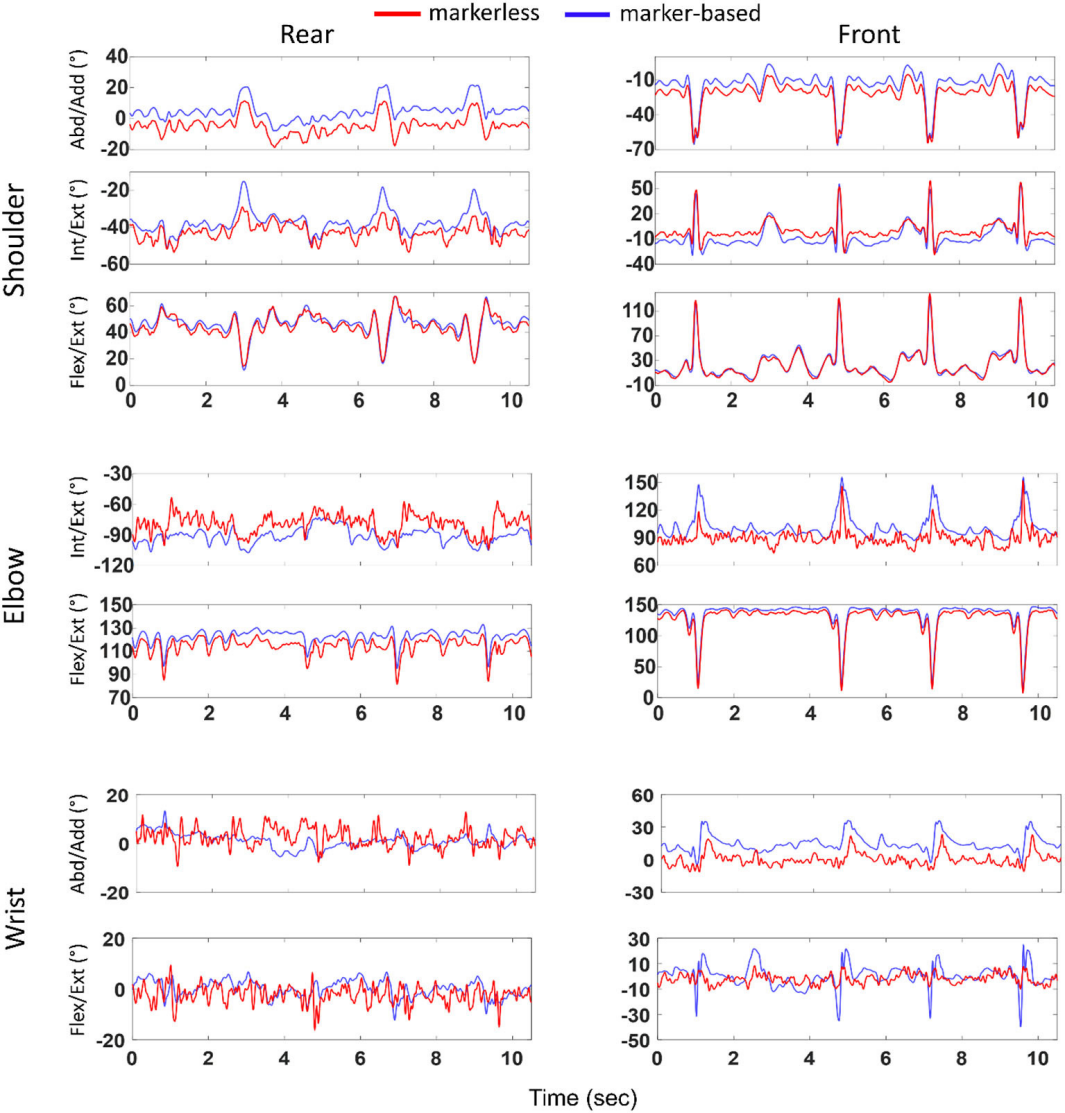
Joint angles (ordinate) at the shoulder, elbow, and wrist computed with marker-based and markerless methods and represented over time (abscissa). Left and right columns represent joints of the rear and front limbs, respectively. Example shown for the second athlete and first boxing trial. Blue and red colors represent marker-based and markerless joint angles, respectively.

Figure 3 illustrates an example (second athlete and first trial) of the joint angles at the shoulder, elbow, and wrist obtained with markerless and marker-based systems. Overall, the kinematic profiles estimated with both the methods exhibited qualitatively similar pattern, with some noticeable offsets.

Table 3 represents the statistical parameters *b, CI*, *R*^2^, and *RMSD* when comparing markerless joint angles with marker-based ones. No values are reported for the elbow and wrist joint along Abd/Add and Int/Ext rotation, respectively, as these DoFs were restricted in the multibody kinematic optimization.

**Table 3:** Bland–Altman bias (*b*), confidence interval (*CI*) along with coefficient of determination (*R*^2^) and root mean square difference (*RMSD*) between markerless and marker-based methods for joint angles at the shoulder, elbow, and wrist.

**Joints**	**Side**	***b***	***CI***	***R* ^2^**	***RMSD***
**Abduction/Adduction (°)**					
Shoulder	Front	3.7	13	0.90	6.6
	Rear	−0.1	15	0.37	6.3
Wrist	Front	7.2	17	0.31	11
	Rear	0.2	21	0.39	9.1
**Internal/External (°)**					
Shoulder	Front	−8.7	9.4	0.83	12
	Rear	2.5	19	0.41	8.1
Elbow	Front	13	30	0.17	23
	Rear	−12	18	0.21	18
**Flexion/Extension (°)**					
Shoulder	Front	2.4	13	0.88	10
	Rear	0.3	8	0.77	7.3
Elbow	Front	−6.2	5.3	0.99	7.4
	Rear	−5.4	8.5	0.87	7
Wrist	Front	7.4	22	0.41	14
	Rear	−0.7	59	0.27	20

*Units for all parameters are in degrees except R^2^ (no unit)*.

Along Abd/Add axis, higher *bias, CI*, and *RMSD*, and lower *R*^2^ values were found at the wrist as compared to the shoulder. Similar outcomes were observed for Int/Ext rotation, with higher *bias, CI*, and *RMSD*, and lower *R*^2^ at the elbow joint. As for the flexion/extension (Flex/Ext) axis, overall, lower *bias* was noticed at the shoulder joint, whereas lower *CI* and *RMSD* were observed at the elbow joint. When comparing across DoFs, highest *bias* and *RMSD* were seen along Int/Ext axis (*bias*: 2.5–13°; *RMSD*: 8.1–23°), followed by Flex/Ext (*bias*: 0.3–7.4°; *RMSD*: 7.3–20°) and Abd/Add (*bias*: −0.1 to 7.2°; *RMSD*: 6.3–11°).

When comparing across joints, lowest *bias* (−0.1°) and lowest *RMSD* (6.3°) were noticed at the shoulder joint, while revealing largest values at the elbow (*bias* up to 13° and *RMSD* up to 23°). Interestingly, between joints on both the sides, lower *bias*, *R*^2^, and *RMSD* were observed at all the rear-side joints compared to its front counterparts with few exceptions.

### Segment velocities

**Figure 4:**
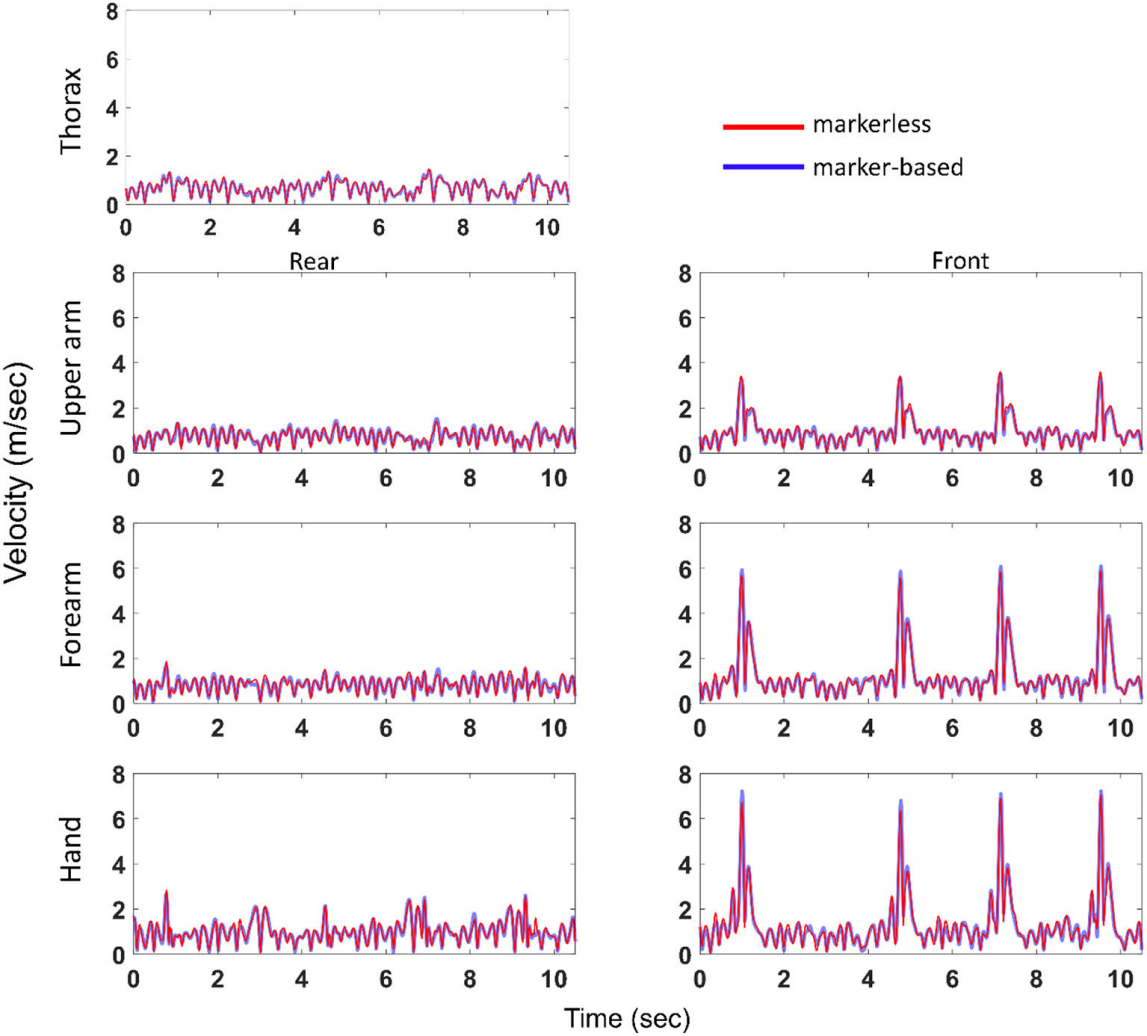
Segment velocity magnitudes (ordinate) at the thorax, and at the front and rear upper arm, forearm, and hand computed with marker-based and markerless methods and represented over time (abscissa). Example shown for the second athlete and first boxing trial. Blue and red colors represent marker-based and markerless segment velocities, respectively.

Figure 4 demonstrates linear segment velocity magnitudes at the thorax, upper arm, forearm, and hand for the second athlete and first trial. The velocity profiles estimated by both the systems displayed qualitatively similar patterns. The median peak velocities across all the athletes and trials measured by the marker-based system were different among segments, with highest velocity of 7.5 m/s at the front hand, followed by the forearm with 5.5 m/s, upper arm with 3.2 m/s, and thorax with 1.6 m/s. These peak velocities were observed while the boxers were throwing punches, and some small velocities (~0–1.5 m/s) were noticed in between punches when they were performing footwork. The markerless system estimated similar results, with 7.0 m/s at the hand, 5.5 m/s at the forearm, 3.5 m/s at the upper arm, and 1.6 m/s at the thorax.

The results of Bland–Altman analysis showed a relatively good agreement between both the systems for the segment velocity magnitudes (Table 4). Very small bias was observed for every segment. Confidence intervals were slightly higher (between 0.10 and 0.25 m/s), but remained small compared to the peak velocity observed during the punch. Overall, the segment with lowest velocity (i.e., thorax) performed the highest level of agreement between both the systems. When comparing between the sides, the rear-side segments showed better agreement as compared to the front-side ones.

**Table 4:** Bland–Altman bias (*b*), confidence interval (*CI*) along with coefficient of determination (*R*^2^) and root mean square difference (*RMSD*) between markerless and marker-based methods for segment velocity magnitudes at the thorax, upper arm, forearm, and hand.

**Velocity magnitudes (m/s)**					
**Segments**	**Side**	***b***	***CI***	***R* ^2^**	***RMSD***
Thorax		0.00	0.12	0.96	0.07
Upper arm	Front	−0.01	0.14	0.98	0.09
	Rear	−0.02	0.13	0.97	0.08
Forearm	Front	−0.03	0.20	0.98	0.14
	Rear	−0.02	0.14	0.98	0.09
Hand	Front	−0.01	0.23	0.98	0.17
	Rear	−0.01	0.17	0.97	0.11

*Units for all parameters are in m/s except R^2^ (no unit)*.

## Discussion

The purpose of the study was to assess whether markerless motion capture system can be exploited to estimate upper-limb kinematics as a substitute to maker-based approach for analyzing sports-performance during “in the field” boxing. We assessed joint center positions, joint angles, and segment velocities obtained with a commercially available markerless motion data processing software (Theia3D) compared to those estimated with classical marker-based method. Multibody models and optimization methods were designed to match at best between the two approaches.

Across all the subjects and trials, the median 3D distances between corresponding joint centers were noticed in the range ~1.5–2.5 cm for all the joints, except the elbow exceeding 3 cm. Our findings were comparable to those, who reported an average difference in the range 1.1–2.4 cm for the upper extremity joints during a treadmill walking activity (Kanko et al., 2021a) and in the range ~2.0–4.7 cm during a throwing activity (Nakano et al., 2020).

The upper limb joint angles captured a varying agreement across all the joints and DoFs. Highest *bias* and *RMSD* were observed along Int/Ext rotation axis and lowest along Abd/Add axis, confirming the remarks reported for the lower extremity joints during gait (Kanko et al., 2021a). However, the values obtained for the upper extremity joints were higher than those obtained for the lower extremity. For instance, *RMSD* along Int/Ext rotation axis was found in the range 6.9–13.2° for the lower extremity (Kanko et al., 2021a), whereas 8.1–23° was observed for the upper extremity in the present work. These higher values could be a consequence of weaker estimation of segment poses during a dynamic activity as compared to gait, respecting the previous evidence of pose estimation performance being task-specific (Nakano et al., 2020; Needham et al., 2021). With regard to all statistical parameters, the shoulder joint demonstrated better agreement between the methods across all DoFs, except Flex/Ext axis along which elbow joint was seen superior. Furthermore, relatively lesser agreement was observed for the front-side joints in general. It is perhaps because of relatively higher and faster movement of the front-side segments resulting higher differences. The front arm is also more often fully extended, a configuration in which determining Int/Ext rotation becomes problematic.

With regard to the segment velocities, the markerless system performed a good-to-strong level of agreement, with maximum *RMSD* ≤ 0.17 m/s and with a strong *R*^2^ (0.96–0.98). Both systems captured highest velocity at the hand (7–7.5 m/s) followed by the other body segments in the kinematic chain. These tendencies corroborate the findings who reported average punch contact velocities in the range 5.9–8.2 m/s for combination of punches (Whiting et al., 1988; Piorkowski et al., 2011).

Overall, we have noticed a higher degree of agreement at the proximal joints/segments between the data collecting methods. This could be a result of the pose algorithm, which may perform less for distal segments, especially for the hand in the considered boxing task as it moves relatively quicker. It could also be a consequence of the multibody kinematic optimization, in which the proximal segments are more constrained than distal segments (they “inherit” the constraints from distal segments) and thus less sensitive to measurement errors. Nevertheless, further investigations are required to confirm these hypotheses.

While interpreting the degree of agreement or differences between the two methods, we would like to highlight few potential sources of errors and assumptions which may influence the results. Marker-based kinematics are prone to misplacement or inconsistent placement of markers. Although the markers were placed by the same operator with adequate palpation skills, some degrees of variability/inconsistency cannot be denied. On the other hand, markerless kinematics are normally susceptible to the quality of 2D video data determined by particularly spatial resolution, exposure time, and angle of view specified for the motion under study. As such we have not studied the sensitivity of these parameters on the kinematic accuracy, we can expect some changes (improvement/deterioration) in the kinematics as reported in other studies (Nakano et al., 2020). Nevertheless, we believe that these impacts would likely to be minimal as data collection was carried out under proficient supervision, and the video data were randomly and qualitatively checked after each acquisition. Furthermore, a repeatability study for the upper extremity seems relevant in the future, although the same has been assessed during gait showing reliable estimation of the lower-limb kinematics using Theia3D (Kanko et al., 2021b). Another source of error commonly known as soft tissue artifact (Camomilla et al., 2017) may impact marker-based kinematics to certain extent, although multibody kinematic optimization was implemented to compensate for it (Begon et al., 2018). Apart from the probable sources of errors, there are some likely differences in defining segment reference frames between the Theia3D kinematic model and marker-based model. For instance, in the marker-based model, the long axis of the thorax is defined between the thoracic joint center and the cervical joint center estimated with regression equations (Dumas and Wojtusch, 2018). Although the Theia3D model uses identical landmarks derived from pose matrices to define the axis, any differences in estimating joint centers would impact the segment frame and thereby resulting in offsets and distortions in the joint angles. We acknowledge that such discrepancies could not be avoided; nevertheless, definition of marker-based joint centers for the shoulder, elbow, and wrist was in accordance with the study carried out by the team involving in Theia3D development (Kanko et al., 2021a,b).

The present work may provide practical avenues to analyze the performance and skill levels of athletes by assessing upper extremity kinematics. For instance, the joint center trajectories and angles have been shown to vary based on the characteristics of boxing type and level of expertise (Whiting et al., 1988; Dinu and Louis, 2020). Measuring these kinematic variables would be necessary to analyze and enhance punches that require a distinct segment orientation in different planes. Information on trajectories of different punch types will also help the combatant to deflect or escape blows (Piorkowski et al., 2011). Furthermore, ranges of motion may provide insights on predisposing factors of injury, as larger joint motion has been reported to implicate joint injury, particularly at the shoulder (Lenetsky et al., 2015). The literature on assessing segment velocities suggests that punch velocity is crucial for optimal performance in boxing (Whiting et al., 1988), with higher values reported for elite boxers (Dinu and Louis, 2020). Attaining high velocity at the fist is typically a result of contribution of other body segments in the kinematic chain. The latter study indicated that the shoulder contributed most to hook and uppercut punch both in junior and in elite boxers. They also noticed moderate differences in segment contribution between high- and low-performing boxers, underlining the need for accurate estimation of segment velocities. Despite such knowledge on the biomechanical distinctions across boxing styles and athletes, receiving timely feedback has been a major burden to coaches and athletes with marker-based methods. In this context, the markerless system is easily deployable both in training sessions and in live combats, while maintaining comparable kinematics to marker-based approach. It is worthwhile to mention that the usability of the kinematic variables as performance measures will depend upon the context of application within an allowable error limit, and this remains an explorable avenue. In marker-based motion analysis, with reference to gait analysis, 5° of error is generally considered the maximum accepted (McGinley et al., 2009). This 5° error correspond to the lower limb, and no such value seems to be present in the literature for the upper limb. That said, we expect segment velocities across all the segments, and joint angles along Abd/Add and Flex/Ext axes can be used with reasonable confidence. The wrist joint angles should be dealt with caution due to relatively poor agreement.

There are few limitations of the present work to acknowledge. We assessed only the upper extremity kinematics, although may not be sufficient to underscore a wide range of performance descriptors such as athletes' stability and kinetic characteristics such as punching force. For example, distribution of the forces between the legs has a considerable effect on punching performance in terms of both stability and fist velocity (Stanley et al., 2018; El-Oujaji et al., 2019). For direct measurement, this would, however, require additional arrangements such as force plates (Piorkowski et al., 2011; Stanley et al., 2018) and instrumented punch bags, making it cumbersome and unsuitable for monitoring “in the field” matches. Studies have also also showed the possibility of estimating punching force using wearable devices and external contact loads (Robert et al., 2013; Muller et al., 2020) using marker-based approach without the need of force sensors. Estimation of these variables using markerless video-based approach seems relevant in assessing sports-performance. One important limitation to highlight is the small number of elite athletes participated in the study. It is also noteworthy to remark that comparative assessment was performed for shadow boxing trials, i.e., one single athlete throwing punches without interaction with the opponent. Although it would be pertinent to analyze the performance of both the athletes in close-combats, evaluating with marker-based motion capture system would be questionable due to its inherent limitations. Moreover, the boxers performed trials without the usage of gloves and boxing outfit as it was convenient to place markers on body landmarks. It would be interesting to analyze the sensitivity of the markerless kinematics in response to traditional boxing attire.

## Conclusion

As a first “in the field” study of a highly dynamic sport, we evaluated 3D joint center positions, joint angles, and segment velocities of the upper extremity of three elite athletes estimated with a markerless approach in comparison with those obtained with marker-based method. We observed a median difference of <2.5 cm for the shoulder and wrist, and slightly higher than 3 cm for the elbow joint between the two approaches. While assessing the joint angles, the shoulder joint largely exhibited a higher level of agreement with RMSD in the range of 6–12°, whereas the wrist and elbow joint displayed more than or equal to 20° in some DoFs. The agreement along the Int/Ext axis was consistently poor across all the DoFs. Segment velocities demonstrated a strong level agreement between the two methods showing a maximum RMSD of 0.17 m/s. Overall results indicated higher levels of agreement between the methods for segment velocities compared to joint angles. Given the practicality of the markerless motion capture system out of the laboratory environment, the results will help both athletes and coaches to analyze sports-performance. Future studies will focus on analyzing both the athletes in close-combat situations with markerless method.

## Data availability statement

The datasets presented in this article are not readily available because of ethical concerns regarding confidentiality. Requests to access the datasets should be directed to thomas.robert@univ-eiffel.fr.

## Ethics statement

The studies involving human participants were reviewed and approved by Local Ethics Committee. The patients/participants provided their written informed consent to participate in this study.

## Author contributions

BL, AM, RD, and TR: design and conceptualization. BL, LR, and TR: data collection. BL: writing—original draft preparation. AM, RD, LR, and TR: writing, reviewing, and editing. TR: supervision. LR: funding. All authors have read and agreed to the submitted version of the manuscript.

## Funding

This study was partly funded by the ANR PPR STHP 2020 (project PerfAnalytics, ANR 20-STHP-0003).

## Conflict of interest

The authors declare that the research was conducted in the absence of any commercial or financial relationships that could be construed as a potential conflict of interest.

## Publisher's note

All claims expressed in this article are solely those of the authors and do not necessarily represent those of their affiliated organizations, or those of the publisher, the editors and the reviewers. Any product that may be evaluated in this article, or claim that may be made by its manufacturer, is not guaranteed or endorsed by the publisher.
