# Supplementary material for: Accuracy of a markerless motion capture system in estimating upper extremity kinematics during boxing
Source: Front Sports Act Living. 2022 Jul 25;4:939980. doi: 10.3389/fspor.2022.939980 (PMC9357930; doi:10.3389/fspor.2022.939980)
Supplement: Supplementary file 1 [file Data_Sheet_1.pdf]

Supplementary Material

1 Supplementary Figures and Tables

1.1 Supplementary Figures

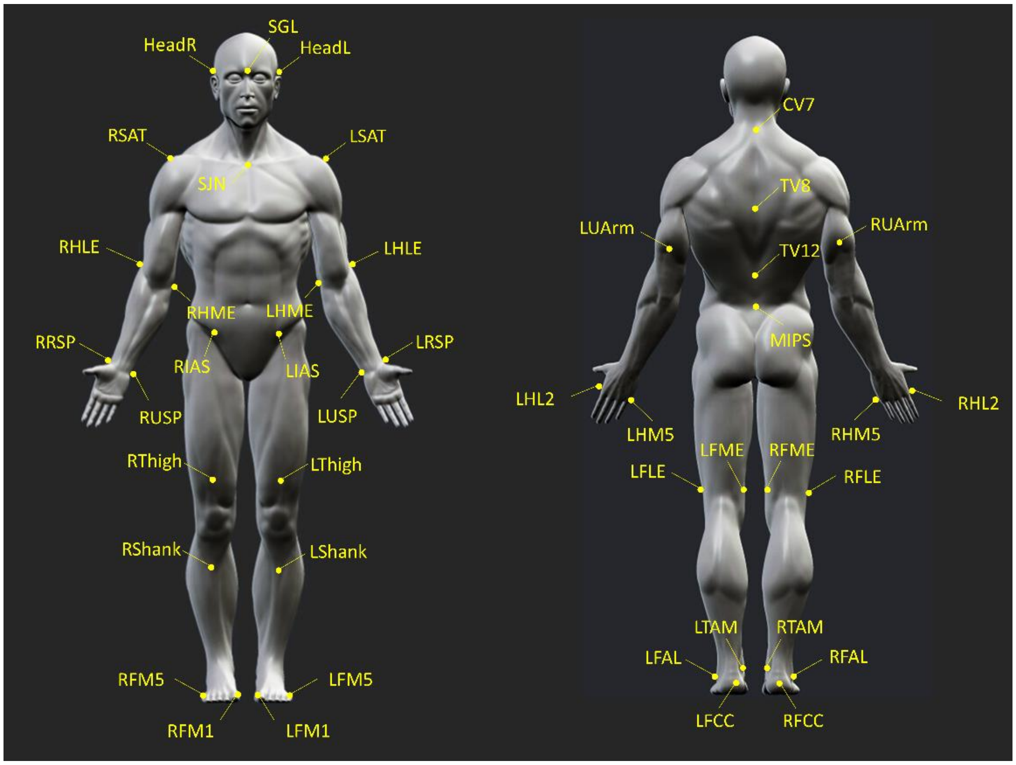

Supplementary Figure 1. Scheme depicting locations of 44 markers on the body (description provided in the supplementary Table 1)

Supplementary Table 2. Marker-set description

Body Region	Acronym	Landmark
**Head**	SGL	Glabella
	HeadR, HeadL	(R, L) Head
**Spine**	CV7	7th Cervical vertebra
	TV8	8th Thoracic vertebra
	TV12	12th Thoracic vertebra
**Sternum**	SJN	Jugular Notch
**Pelvis**	RIAS, LIAS	(R, L) Anterior Superior Iliac Spine
	MIPS	Mid of Posterior Superior Iliac Spines
**Upper-arm**	RSAT, LSAT	(R, L) Scapula-Acromial Tip
	RHLE, LHLE	(R, L) Humerus Lateral Epicondyle
	RHME, LMLE	(R, L) Humerus Medial Epicondyle
	RUArm, LUArm	(R, L) Upper Arm
**Forearm**	RRSP, LRSP	(R, L) Radius Styloid Process
	RUSP, LUSP	(R, L) Ulna Styloid Process
**Hand**	RHL2, LHL2	(R, L) 2nd Metacarpal Head Lateral
	RHM5, LHM5	(R, L) 5th Metacarpal Head Medial
**Thigh**	RFLE, LFLE	(R, L) Femoral Lateral Epicondyle
	RFME, LFME	(R, L) Femoral Medial Epicondyle
	RThigh, LThigh	(R, L) Thigh
**Shank**	RFAL, LFAL	(R, L) Apex of Lateral Malleolus
	RTAM, LTAM	(R, L) Apex of Medial Malleolus
	RShank, LShank	(R, L) Shank
**Foot**	RFCC, LFCC	(R, L) Foot Calcaneus Center
	RFM1, LFM1	(R, L) 1st Foot Metatarsus
	RFM5, LFM5	(R, L) 5th Foot Metatarsus
